# Supplementary material for: Cryo-EM structures of S-OPA1 reveal its interactions with membrane and changes upon nucleotide binding
Source: eLife. 2020 Mar 31;9:e50294. doi: 10.7554/eLife.50294 (PMC7156267; doi:10.7554/eLife.50294)
Supplement: Supplementary file 1. — The raw data come from three independent experiments. [file elife-50294-supp1.docx]

**Table S1. Enzymatic Km and Kcat of S-OPA1 and its mutants. The data presented come from three independent experiments (Data S1).**

|  | No liposome | | | | With liposome | | | |
| --- | --- | --- | --- | --- | --- | --- | --- | --- |
|  | Km (mM) | Stdev | Kcat (1/min) | Stdev | Km (mM) | Stdev | Kcat (1/min) | Stdev |
| WT | 1.2 | 0.7 | 3.9E-04 | 1.5E-04 | 5.0 | 5.5 | 2.3E-02 | 2.2E-02 |
| Δ196-252 | 0.4 | 0.2 | 7.2E-05 | 1.8E-05 | 0.8 | 0.6 | 1.1E-02 | 4.5E-03 |
| Q297E | 0.5 | 0.2 | 1.2E-04 | 1.8E-05 | 9.4 | 14.1 | 2.1E-03 | 3.0E-03 |
| S298A | 0.4 | 0.1 | 1.2E-04 | 1.2E-05 | 0.6 | 0.3 | 2.1E-04 | 5.6E-05 |
| G300E | 0.9 | 0.4 | 1.5E-04 | 3.9E-05 | 0.6 | 0.5 | 1.5E-04 | 5.7E-05 |
| T302N | 0.7 | 0.1 | 2.2E-04 | 1.7E-05 | 1.1 | 0.3 | 4.2E-04 | 6.6E-05 |
| T323A | 0.9 | 0.2 | 1.8E-04 | 2.6E-05 | 2.4 | 1.1 | 7.8E-04 | 2.8E-04 |
| 794-800A | 0.6 | 0.2 | 2.2E-04 | 3.0E-05 | 1.1 | 0.3 | 9.1E-04 | 1.6E-04 |
| E794AE796A | 0.5 | 0.1 | 5.3E-04 | 4.1E-05 | 2.8 | 10.4 | 5.3E-03 | 1.5E-02 |
| K797AK800A | 0.5 | 0.5 | 2.2E-04 | 9.1E-05 | 1.5 | 0.6 | 9.4E-03 | 2.7E-03 |
| L795EM798EL799E | 1.9 | 1.1 | 3.8E-04 | 1.7E-04 | 1.1 | 0.6 | 3.9E-04 | 1.4E-04 |
| L795AM798AL799A | 0.9 | 0.2 | 3.6E-04 | 4.1E-05 | 1.3 | 0.2 | 1.1E-03 | 1.4E-04 |
