# Supplementary material for: Cryo-EM structures of S-OPA1 reveal its interactions with membrane and changes upon nucleotide binding
Source: eLife. 2020 Mar 31;9:e50294. doi: 10.7554/eLife.50294 (PMC7156267; doi:10.7554/eLife.50294)
Supplement: Supplementary file 2. — Data was obtained from UniProt (https://www.uniprot.org). [file elife-50294-supp2.docx]

**Table S2. Disease related mutants in S-OPA1. Data was obtained from UniProt (**[**https://www.uniprot.org)**](https://www.uniprot.org))**.**

| **Optic atrophy 1 (OPA1)** | | | | |
| --- | --- | --- | --- | --- |
| Position(s) | Mutant | Domain (Predicted) | Description | Reference |
| 8 | A → S | G domain | Unknown pathological significance. | (1) |
| 38 – 43 | Missing | G domain |  | (2) |
| 80 | Y → C | G domain |  | (1) |
| 95 | T → M | G domain |  | (3) |
| 102 | Y → C | G domain |  | (3) |
| 270 | E → K | G domain |  | (4) |
| 272 | L → P | G domain |  | (5) |
| 273 | D → A | G domain |  | (4) |
| 290 | R → Q | G domain |  | (4, 6-8) |
| 290 | R → W | G domain |  | (4) |
| 293 – 294 | Missing | G domain |  | (3) |
| 300 | G → E | G domain | Loss of GTPase activity; loss of function in promoting mitochondrial fusion. | (8-10) |
| 310 | Q → R | G domain |  | (3) |
| 324 – 326 | Missing | G domain |  | (11) |
| 330 | T → S | G domain |  | (12) |
| 357 | A → T | G domain | In DOA+ and OPA1. | (3, 13) |
| 377 | V → I | G domain |  | (12) |
| 382 | I → M | G domain | In OPA1 and BEHRS. | (3, 14, 15) |
| 384 | L → F | G domain |  | (8) |
| 396 | L → P | G domain |  | (3) |
| 396 | L → R | G domain |  | (2) |
| 400 | P → A | G domain |  | (16) |
| 429 – 430 | Missing | G domain |  | (3) |
| 430 | N → D | G domain |  | (3) |
| 432 | Missing | G domain |  | (2, 7) |
| 438 | D → V | G domain |  | (4) |
| 439 | G → V | G domain | In DOA+ and OPA1; decreased GTPase activity; loss of function in promoting mitochondrial fusion. | (10, 13, 17) |
| 445 | R → H | G domain | In DOA+ and OPA1; decreased GTPase activity; loss of function in promoting mitochondrial fusion. | (10, 13, 18-20) |
| 449 | T → R | G domain |  | (3) |
| 459 | G → E | G domain |  | (21) |
| 463 | I → IFIF | G domain |  |  |
| 468 | K → E | G domain |  | (4) |
| 470 | D → G | G domain |  | (5) |
| 487 | E → K | G domain | In OPA1 and BEHRS. | (3, 14) |
| 503 | T → K | G domain |  | (2, 8) |
| 505 | K → N | G domain |  | (8) |
| 545 | S → R | Middle | In DOA+ and OPA1; decreased GTPase activity; loss of function in promoting mitochondrial fusion. | (3, 10, 13, 18, 22) |
| 551 | C → Y | Middle | In OPA1 and DOA+. | (3, 23) |
| 551 | Missing | Middle |  | (4) |
| 571 | R → H | Middle |  | (2) |
| 574 | L → P | Middle |  | (5) |
| 586 – 589 | Missing | Middle |  | (2) |
| 590 | R → Q | Middle |  | (3) |
| 590 | R → W | Middle |  | (11) |
| 593 | L → P | Middle |  | (3) |
| 593 | Missing | Middle |  | (24) |
| 646 | S → L | Middle |  | (3) |
| 700-701 | Missing | EMB |  | (5) |
| 728 | N → K | EMB | Loss of function in promoting mitochondrial fusion. | (10, 11) |
| 768 | G → D | EMB |  | (3) |
| 781 | R → W | EMB |  | (3) |
| 785 | Q → R | EMB | Loss of lipid binding and partial loss of function in promoting mitochondrial fusion. | (4, 6, 10) |
| 823 | S → Y | EMB |  | (3) |
| 841 | Y → C | EMB |  | (1) |
| 882 | R → L | EMB |  | (3) |
| 887 | L → P | EMB |  | (3) |
| 910 | Missing | EMB |  | (21) |
| 932 | R → C | EMB |  | (3, 25) |
| 939 | L → P | EMB | Impairs protein folding; loss of function in promoting mitochondrial fusion. | (6, 10) |
| 949 | L → P | EMB |  | (3, 18) |
| **Dominant optic atrophy plus syndrome (DOA+)** | | | | |
| 357 | A → T | G domain | In DOA+ and OPA1. | (3, 13) |
| 439 | G → V | G domain | In DOA+ and OPA1; decreased GTPase activity; loss of function in promoting mitochondrial fusion | (10, 13, 17) |
| 445 | R → H | G domain | In DOA+ and OPA1; decreased GTPase activity; loss of function in promoting mitochondrial fusion | (10, 13, 18-20) |
| 449 | T → P | G domain |  | (26) |
| 545 | S → R | Middle | In DOA+ and OPA1; decreased GTPase activity; loss of function in promoting mitochondrial fusion. | (3, 10, 13, 18, 22) |
| 551 | C → Y | Middle | In OPA1 and DOA+. | (3, 23) |
| 582 | Y → C | Middle |  | (26) |
| 910 | V → D | GED | Impairs protein folding; loss of function in promoting mitochondrial fusion. | (10, 13) |
| **Behr syndrome (BEHRS)** | | | | |
| 382 | I → M | G domain | In OPA1 and BEHRS | (3, 14, 15) |
| 402 | V → M | G domain | In BEHRSl. | (27) |
| 487 | E → K | G domain | In OPA1 and BEHRS. | (3, 27) |
| **Mitochondrial DNA depletion syndrome 14, cardioencephalomyopathic type (MTDPS14)** | | | | |
| 534 | L → R | Middle |  | (28) |

**References:**

1. Han J*, et al.* (2006) OPA1 mutations and mitochondrial DNA haplotypes in autosomal dominant optic atrophy. *Genet Med* 8(4):217-225.

2. Thiselton DL*, et al.* (2002) A comprehensive survey of mutations in the OPA1 gene in patients with autosomal dominant optic atrophy. *Invest Ophthalmol Vis Sci* 43(6):1715-1724.

3. Ferre M*, et al.* (2009) Molecular screening of 980 cases of suspected hereditary optic neuropathy with a report on 77 novel OPA1 mutations. *Hum Mutat* 30(7):E692-705.

4. Pesch UE*, et al.* (2001) OPA1 mutations in patients with autosomal dominant optic atrophy and evidence for semi-dominant inheritance. *Hum Mol Genet* 10(13):1359-1368.

5. Baris O*, et al.* (2003) Fourteen novel OPA1 mutations in autosomal dominant optic atrophy including two de novo mutations in sporadic optic atrophy. *Hum Mutat* 21(6):656.

6. Delettre C*, et al.* (2001) Mutation spectrum and splicing variants in the OPA1 gene. *Hum Genet* 109(6):584-591.

7. Alexander C*, et al.* (2000) OPA1, encoding a dynamin-related GTPase, is mutated in autosomal dominant optic atrophy linked to chromosome 3q28. *Nat Genet* 26(2):211-215.

8. Toomes C*, et al.* (2001) Spectrum, frequency and penetrance of OPA1 mutations in dominant optic atrophy. *Hum Mol Genet* 10(13):1369-1378.

9. Delettre C*, et al.* (2000) Nuclear gene OPA1, encoding a mitochondrial dynamin-related protein, is mutated in dominant optic atrophy. *Nat Genet* 26(2):207-210.

10. Ban T, Heymann JA, Song Z, Hinshaw JE, & Chan DC (2010) OPA1 disease alleles causing dominant optic atrophy have defects in cardiolipin-stimulated GTP hydrolysis and membrane tubulation. *Hum Mol Genet* 19(11):2113-2122.

11. Puomila A*, et al.* (2005) Dominant optic atrophy: correlation between clinical and molecular genetic studies. *Acta Ophthalmol Scand* 83(3):337-346.

12. Chen Y*, et al.* (2013) Mutation survey of the optic atrophy 1 gene in 193 Chinese families with suspected hereditary optic neuropathy. *Mol Vis* 19:292-302.

13. Amati-Bonneau P*, et al.* (2008) OPA1 mutations induce mitochondrial DNA instability and optic atrophy 'plus' phenotypes. *Brain* 131(Pt 2):338-351.

14. Schaaf CP*, et al.* (2011) Early-onset severe neuromuscular phenotype associated with compound heterozygosity for OPA1 mutations. *Mol Genet Metab* 103(4):383-387.

15. Carelli V*, et al.* (2015) 'Behr syndrome' with OPA1 compound heterozygote mutations. *Brain* 138(Pt 1):e321.

16. Zhang J*, et al.* (2012) A novel OPA1 mutation in a Chinese family with autosomal dominant optic atrophy. *Biochem Biophys Res Commun* 419(4):670-675.

17. Liguori M*, et al.* (2008) A phenotypic variation of dominant optic atrophy and deafness (ADOAD) due to a novel OPA1 mutation. *J Neurol* 255(1):127-129.

18. Nakamura M*, et al.* (2006) Novel mutations in the OPA1 gene and associated clinical features in Japanese patients with optic atrophy. *Ophthalmology* 113(3):483-488 e481.

19. Payne M*, et al.* (2004) Dominant optic atrophy, sensorineural hearing loss, ptosis, and ophthalmoplegia: A syndrome caused by a missense mutation in OPA1. *American Journal of Ophthalmology* 138(5):749-755.

20. Amati-Bonneau P*, et al.* (2005) OPA1 R445H mutation in optic atrophy associated with sensorineural deafness. *Ann Neurol* 58(6):958-963.

21. Almind GJ*, et al.* (2012) Dominant optic atrophy in Denmark - report of 15 novel mutations in OPA1, using a strategy with a detection rate of 90%. *BMC Med Genet* 13:65.

22. Hudson G*, et al.* (2008) Mutation of OPA1 causes dominant optic atrophy with external ophthalmoplegia, ataxia, deafness and multiple mitochondrial DNA deletions: a novel disorder of mtDNA maintenance. *Brain* 131(Pt 2):329-337.

23. Marelli C*, et al.* (2011) Heterozygous OPA1 mutations in Behr syndrome. *Brain* 134(Pt 4):e169; author reply e170.

24. Yen MY, Wang AG, Lin YC, Fann MJ, & Hsiao KJ (2010) Novel mutations of the OPA1 gene in Chinese dominant optic atrophy. *Ophthalmology* 117(2):392-396 e391.

25. Nochez Y*, et al.* (2009) Acute and late-onset optic atrophy due to a novel OPA1 mutation leading to a mitochondrial coupling defect. *Mol Vis* 15:598-608.

26. Ferraris S*, et al.* (2008) Progressive external ophthalmoplegia and vision and hearing loss in a patient with mutations in POLG2 and OPA1. *Arch Neurol* 65(1):125-131.

27. Bonneau D*, et al.* (2014) Early-onset Behr syndrome due to compound heterozygous mutations in OPA1. *Brain* 137.

28. Spiegel R*, et al.* (2016) Fatal infantile mitochondrial encephalomyopathy, hypertrophic cardiomyopathy and optic atrophy associated with a homozygous OPA1 mutation. *J Med Genet* 53(2):127-131.
